# Optimal Control of Hepatitis C Antiviral Treatment Programme Delivery for Prevention amongst a Population of Injecting Drug Users

**DOI:** 10.1371/journal.pone.0022309

**Published:** 2011-08-11

**Authors:** Natasha K. Martin, Ashley B. Pitcher, Peter Vickerman, Anna Vassall, Matthew Hickman

**Affiliations:** 1 Department of Social Medicine, University of Bristol, Bristol, United Kingdom; 2 Department of Global Health and Development, London School of Hygiene and Tropical Medicine, London, United Kingdom; 3 Centre d'Analyse et de Mathématique Sociales, L'École des Hautes Études en Sciences Sociales, Paris, France; Erasmus University Rotterdam, The Netherlands

## Abstract

In most developed countries, HCV is primarily transmitted by injecting drug users (IDUs). HCV antiviral treatment is effective, and deemed cost-effective for those with no re-infection risk. However, few active IDUs are currently treated. Previous modelling studies have shown antiviral treatment for active IDUs could reduce HCV prevalence, and there is emerging interest in developing targeted IDU treatment programmes. However, the optimal timing and scale-up of treatment is unknown, given the real-world constraints commonly existing for health programmes. We explore how the optimal programme is affected by a variety of policy objectives, budget constraints, and prevalence settings. We develop a model of HCV transmission and treatment amongst active IDUs, determine the optimal treatment programme strategy over 10 years for two baseline chronic HCV prevalence scenarios (30% and 45%), a range of maximum annual budgets (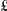
50,000–300,000 per 1,000 IDUs), and a variety of objectives: minimising health service costs and health utility losses; minimising prevalence at 10 years; minimising health service costs and health utility losses with a final time prevalence target; minimising health service costs with a final time prevalence target but neglecting health utility losses. The largest programme allowed for a given budget is the programme which minimises both prevalence at 10 years, and HCV health utility loss and heath service costs, with higher budgets resulting in greater cost-effectiveness (measured by cost per QALY gained compared to no treatment). However, if the objective is to achieve a 20% relative prevalence reduction at 10 years, while minimising both health service costs and losses in health utility, the optimal treatment strategy is an immediate expansion of coverage over 5–8 years, and is less cost-effective. By contrast, if the objective is only to minimise costs to the health service while attaining the 20% prevalence reduction, the programme is deferred until the final years of the decade, and is the least cost-effective of the scenarios.

## Introduction

Hepatitis C virus (HCV) is a comparatively common blood-borne disease with 130–170 million people (2–3%) globally infected [1]. It is one of the leading causes of chronic liver disease worldwide, and is the fastest growing cause of liver transplantation in developed countries [2]. If left untreated, about 7–18% progress to liver disease within 20 years, which can result in liver failure, cirrhosis, hepatocellular carcinoma, and death [3].

The primary mode of transmission in developed countries is amongst active injecting drug users (IDUs) where it is easily transmitted through needle and syringe sharing. In the UK, most developed countries, and many other developing countries without marked iatrogenic HCV risk (such as South Asia), over 80% of new cases are attributed to injecting drugs, with 15–90% of IDUs testing positive for HCV antibodies [4]–[7]. Public health interventions such as health education and advice, needle and syringe exchange, and opiate substitution therapy aim to prevent transmission by reducing unsafe injecting [4]. However, despite increases in intervention exposure, public health surveillance indicates that substantial decreases in HCV prevalence have not been achieved [8].

Antiviral treatment (peginterferon-alfa and ribavirin) for HCV has been established as effective and results in viral clearance in about 45–80% of cases, depending on genotype [9]–[11]. Economic evaluations have found treatment cost-effective for a population with no re-infection risk [12]. Since 2002, guidelines in the US and UK do not exclude active IDUs from treatment eligibility, given the growing evidence that IDUs exhibit a similar response to treatment, and could be just as compliant with treatment as ex- or non-IDUs [11]–[13]. Despite these recommendations and the high proportion of IDUs infected, very few (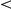
3–4%) active IDUs have ever been treated [14], [15]. Recent mathematical modelling has predicted that antiviral treatment can be an effective prevention measure amongst IDUs, with modest and achievable levels of treatment resulting in substantial reductions in infected prevalence [16]–[19]. Hence, treatment of injectors could have substantial benefits in relation to reducing ongoing transmission (despite some of the difficulties in delivering the treatment and potential loss of sustained viral response (SVR)).

Ideally, from an economic perspective, any intervention, such as HCV treatment, that has been found to be cost-effective would be fully funded immediately. In reality, however, funding and access to treatment may not be provided as programmes are always faced with a number of constraints. Globally, there are many interventions that fall under the WHO willingness to pay thresholds, but remain underfinanced by countries and face substantial budget constraints. For example, in Australia free HCV treatment is available under the national health care system, but the government acknowledges a capacity limit within the specialist hepatitis C treatment services which restricts the numbers that can undergo treatment each year [20]. In the UK, NICE (National Institute for Health and Clinical Excellence) issues guidelines on which treatments should be offered, but different regions frequently offer different levels of treatment. Global (and sometimes national) institutions frequently respond to this issue by setting targets, either for coverage or to achieve specific reductions in prevalence. Indeed, the low coverage of HCV treatment across the UK has resulted in the development of a number of national action plans (Scotland, Wales, and England) which aim to expand treatment coverage over the next 5 years [20]–[22]. These action plans do not specifically target current IDUs, despite the growing interest in targeting antiviral treatment to IDUs as a means of prevention, and general movements for more active IDUs to be treated [20], [23]. This study examines how constraints (annual maximum budgets) and objectives (prevalence targets, desire to minimise health utility losses) are likely to influence optimal timing and intensity of scale-up, and the subsequent costs, impact, and cost per QALY (quality-adjusted life year) gained in each scenario.

As such, our aim is not to perform an economic evaluation of antiviral treatment, but to inform policy makers on how the optimal treatment strategy and programme cost-effectiveness (measured by cost per QALY gained) may be affected when different constraints are applied. This type of analysis (using optimal control theory to determine the optimal resource allocation as an epidemic progresses) has been used before in infectious disease prevention [24]–[27]. Hence, our aims and mathematical techniques are well established in infectious disease literature, although its specific application to HCV is novel. Importantly, few have managed to present this technique to a broader audience (outside of mathematical modellers) and with real-world budget constraints and objectives.

We parameterise our epidemic model with recent data from the UK, and our cost coefficients with current UK costs of antiviral treatment and HCV infection. We then examine the optimal treatment strategy for different economic and policy objectives, which range from ‘ideal’ public health objectives (where health service costs and HCV health utility loss or just prevalence is minimised) to ‘less ideal’ but perhaps more realistic scenarios with a specific policy objective of reducing prevalence by a specific percentage by the end of the 10 year timeframe. The specific scenarios we examine are: 1) minimising health service costs and health utility (QALY) loss; 2) minimising prevalence; 3) minimising health service costs and health utility loss while achieving a final time prevalence reduction of 20%; 4) minimising health service costs while achieving a final time prevalence reduction of 20% (and neglecting health utility loss). This is done for a variety of annual budget constraints and two baseline prevalences (30% and 45%).

## Methods

### Model background and assumptions

Infection with HCV leads to a relatively short (weeks to months) acute stage, which may lead to a prolonged chronic stage lasting for decades [28]. A fraction (about 26%) of acute infections are spontaneously cleared by the individual [29]. Due to the short duration of the acute stage, the number of infections caused by people with acute HCV who spontaneously clear is small, and we neglect it for model simplicity. Those who spontaneously clear become susceptible again, and the remaining fraction who do not spontaneously clear progress to the chronic infection stage. There is controversy around the possibility of sterilising immunity following exposure to HCV. However, given that immunity following exposure to HCV is uncertain, and previous models have shown that, if present, this population is relatively small, we neglect it for the purposes of this model [16], [17].

Antiviral treatment leads to a substantial reduction in viral load in the first few weeks (even among some eventual nonresponders) [30]. Hence, we assume that active IDUs currently on treatment are non-infectious. Due to the lack of definitive evidence to suggest otherwise, we assume that the chances of spontaneous clearance are equal for naïve (those who have never been infected) and re-infected IDUs. Furthermore, we assume that the probability of treatment success is the same between naïve and re-infecteds, which is supported by experimental evidence [31]. Finally, we assume that treatment failures return to the chronically infected population and are eligible for retreatment as a simplifying assumption, as alternative dosing and treatment durations are available for this group [32]. Nonetheless, over our relatively short timescale (10 years) and with the low level of treatment examined in this manuscript, simulations tracking nonresponders show that negligible levels of infecteds are retreated [16].

### Details and explanation of the model

We model the transmission of HCV amongst active IDUs, using a system of ordinary differential equations simplified from [16], [17]. We utilise a three compartment model, tracking susceptible, chronically infected, and treated IDUs. Susceptible IDUs become infected through sharing needles with infected IDUs. About one quarter spontaneously clear the infection, and become susceptible again. The remaining three-quarters progress to chronic infection. Chronic infecteds can be treated, with a certain chance of success, and either fail treatment and return to the infection compartment, or clear the disease and become susceptible again.

In our model, 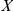 denotes the number of susceptible IDUs (including those who have cleared the infection), 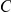 denotes the number of both chronically infected and acutely infected IDUs which will proceed to chronic infection, and 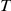 denotes the number of IDUs in treatment, 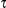 is time in years, and where 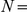
total population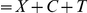. The equations describing the HCV transmission are:
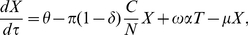
(1)

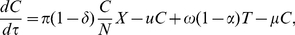
(2)

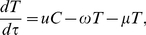
(3)with initial conditions 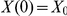, 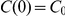, 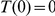. For the initial conditions, we assume that the epidemic is at steady state with no treatment, as is the case in most places in the UK [8].

Equation (1) represents the rate of change of the size of the susceptible population, where new IDUs enter at a fixed rate 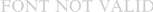. The second term in Equation (1) models the infection of susceptible IDUs, which is proportional to the number of susceptibles, the fraction of the population chronically infected, and the infection rate 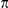. The acute infection spontaneously clears in a proportion 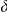, who return to the susceptible pool. The remaining infected fraction which do not spontaneously clear, 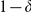, progress to chronic infection. The third term in Equation (1) represents IDUs who exit treatment at a rate 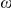, with successful treatment proportion 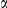.

Equation (2) models the rate of change of the number of chronically infected IDUs. The first term represents those who enter from the susceptible pool, which is proportional to the number of susceptibles, the fraction of the population chronically infected, the infection rate 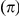, and the fraction who do not spontaneously clear the acute infection 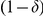. The fraction of nonresponders to treatment, 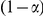, return from treatment proportional to rate 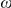.

The second term in Equation (2), 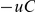, represents the movement of infected IDUs into treatment. The proportion of infecteds put on treatment per year as an instantaneous rate represented by 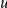, can vary through time and is the function we would like to optimise with respect to, given the constraints described later.

Equation (3) represents the rate of change of the number of IDUs currently in treatment. Infected IDUs enter treatment at the rate 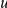 as discussed for Equation (2). IDUs exit treatment proportional to the rate 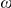.

In each of the populations, IDUs leave (due to death or ceasing injection) proportional to the rate 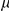.

### System and Objective Functional

If we let 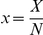, 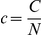, 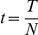, so that the state variables are now the fraction of the population in each compartment, then Equations (1)–(3) become
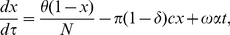
(4)

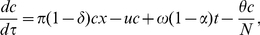
(5)

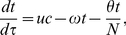
(6)where 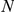 evolves according to the equation
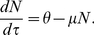
(7)Since the population is assumed to be in steady state, then 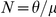. Making this substitution, Equations (4)–(6) become
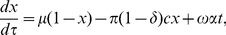
(8)

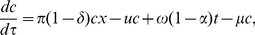
(9)

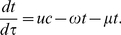
(10)Since 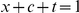, we can substitute 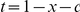 into Equations (8) and (9) and get rid of (10) so that we are left with the smaller system
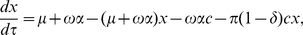
(11)

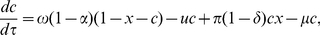
(12)with initial conditions
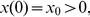
(13)

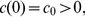
(14)where 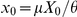, 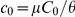 and 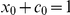. We wish to find the function 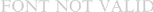 over the time horizon 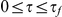 such that we minimise the objective functional
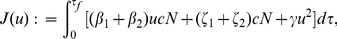
(15)subject to the constraints
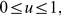
(16)

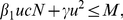
(17)where 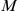 is a positive constant representing the maximum programme spend rate, and 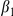, 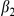, 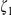, 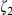 and 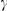 are the following decreasing functions of time:
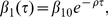
(18)

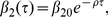
(19)

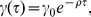
(20)

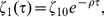
(21)

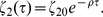
(22)The parameters 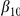, 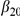, 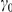, 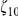, 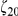 and 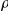 are positive constants. The constant 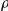 is the instantaneous discount rate and can be calculated using the formula
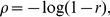
(23)where 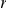 is the annual effective discount rate.

We divided the cost associated with HCV into two types. First, we have the programme costs. These are the costs required to search for and treat patients. Secondly, we have the costs associated with infection. These are the costs incurred by the health service when managing patients who are untreated and remain infected. Together, programme (antiviral treatment and search) and infection costs add up to the health service costs associated with HCV. More precisely, the function 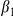 represents the average cost of antiviral treatment per person. The search costs related to finding, diagnosing, and recruiting active IDUs onto treatment are represented by the term 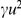. As is commonly done in optimal control models of infectious diseases, we use a quadratic function of the control to represent increasing marginal costs associated with achieving high treatment coverage levels [24], [33]. The function 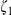 represents the average annual infection cost per chronically infected IDU.

To be able to incorporate health utility losses into the optimal control framework, we monetarise the QALY losses associated with HCV infection and antiviral treatment. We detail this approach fully in the section ‘Economic parameters’. In particular, 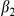 represents the monetarisation of the QALY loss per antiviral treatment, and 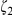 represents the monetarisation of the QALY loss per year associated with HCV. The parameters 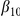, 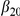, 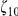, and 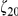 represent the initial values of these costs at 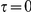.

The first constraint 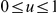 means that we cannot treat at a rate higher than 100% of the infected population per year, and the treatment rate cannot be negative. The second constraint 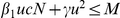 limits the spending rate of the treatment programme (antiviral treatment and search costs) so that the programme spending rate can never be greater than 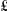 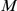 per year. 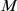 will be referred to as the annual or yearly budget. As health service costs related to infection, 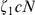, are unrelated to the direct programme budget, we do not incorporate these into the budget constraint.

With our model, we project the optimal treatment programme over time, the corresponding prevalence reductions and infections averted with each programme, total programme costs, and total infection costs. We divide our total programme costs by the number of infections averted to calculate cost per infection averted. We also calculate net monetary benefit (monetarised benefits associated with QALYs gained minus net health services costs) related to each programme compared to no treatment. Finally, we calculate the health service cost per QALY gained for each programme. Due to the prevention impact of antiviral treatment, it is important to capture the future benefits of the treatment programme. For these calculations, we simulate each 10 year treatment programme, and then continue calculating discounted health service costs and QALYs for a further 40 years, in order to determine the onward prevention impact of the different 10 year treatment programmes. Hence, the total time horizon in the cost per QALY gained calculation is 50 years. We calculate the cost per QALY gained as compared to a baseline of no treatment.

### Optimal control

The optimal control problem is 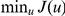 subject to (11)–(14), (16) and (17). In other words, we seek the function 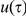 such that 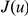 is minimised subject to the state equations (11) and (12), the initial conditions (13) and (14) and the constraints (16) and (17).

We employ Pontryagin's minimum principle [34] to determine necessary conditions that must be satisfied by an optimal control, if one exists. The existence of an optimal control and corresponding optimal states is guaranteed for this optimal control problem as criteria (a),(b),(c) of Theorem 4.1 and criteria (d') and (e') of Corollary 4.1 found in [35, chap.3] are satisfied.

The optimal control theory associated with various types of inequality constraints, including the ones considered here, can be found in [36]. For a general introductory text on optimal control methods applied to biological systems, we direct interested readers towards the text by Lenhart and Workman [37]. The first step is to form the Hamiltonian associated with this optimal control problem, i.e.
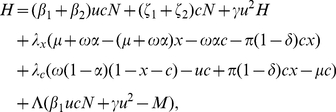
(24)where 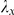, 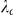 and 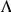 are functions of 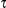. The multiplier 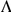 is an adjoining Lagrange multiplier that must satisfy
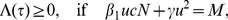
(25)

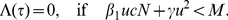
(26)To find the characterisation of the optimal control, by Pontryagin's minimum principle we just need to find the 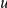 that minimises the Hamiltonian. Since the control 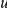 appears quadratically in the Hamiltonian, then to do this we simply set
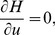
(27)which gives the equation
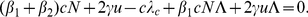
(28)If 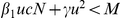, then (28), with 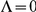 and the constraint (16), provides the optimal control characterisation (call it 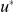):
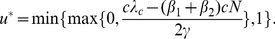
(29)If 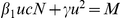, then the optimal control characterisation is derived by simply solving this quadratic equation for 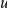 to obtain
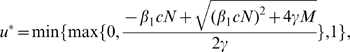
(30)where we have taken the positive root because the negative root always yields a negative value of the fraction in (30) since the parameters are all positive. Due to the control constraint (16), the optimal control is forced to lie within its admissible bounds 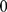 and 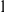.

We know from (26) that 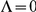 when the constraint (17) is not tight. When the budget constraint is tight, the function 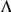 can be determined from (28) with 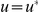 as defined in (30), so that
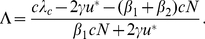
(31)


It is important to keep in mind that the search cost coefficient 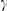 cannot at anytime be zero or else the optimal control problem is singular. Singular optimal control problems require different solution methods (see [38]–[40], and [37]).

The corresponding differential equations for the adjoint variables (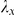 and 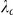) are determined from partial derivatives of the Hamiltonian:
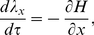
(32)

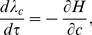
(33)from which we obtain the adjoint equations
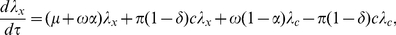
(34)

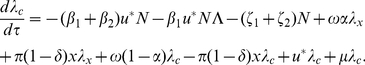
(35)The adjoint equations have the final conditions
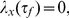
(36)

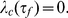
(37)


The state and adjoint equations and their four associated boundary conditions constitute a two-point boundary value problem which we solve in MATLAB using the *forward-backward sweep* numerical method [37].

In order to force a specific final time prevalence, we must remove the final time condition 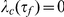. Instead, an iterative bisection method is used to find the value of 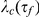 that yields the desired final time target prevalence 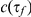.

Numerical solutions with a final time prevalence specified are run with 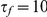 years. Numerical solutions without a final time prevalence specified are run with 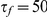 years, and results presented for the first 10 years. The use of an expanded time range (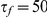) when there is no final time prevalence specified is necessary to ensure that premature termination of treatment is not recommended due to the lack of consideration for onward transmission after the initial decade of interest. As new treatments will likely be introduced in the next decade (with different cost implications), it is reasonable to focus on a 10 year timeframe.

If instead we wish to simply minimise final time prevalence subject to our budget constraint, we can do this by adding the term 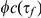 to the objective functional and making the positive constant 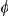 very large, e.g. 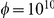 so that the other terms in the objective are negligible by comparison. Mathematically, this addition to the objective functional leads to a different final time boundary condition on 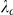, i.e. 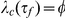.

### Uncertainty analysis

To examine how the uncertainty in the biological and cost parameters alters the optimal control solution, we perform a Latin Hypercube Sampling (LHS) of the cost parameters (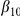, 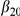, 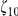, 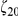, 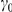) and biological parameters (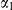, 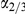, 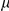 and 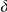) over a uniform range of values. For each of the 100 parameter sets in our sampling, we calculate the corresponding infection rate (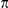) and new injector rate (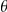) which gives the desired untreated endemic prevalence (30% or 45%), and retains a total of 1,000 IDUs in the population. With each of these parameter sets, we calculate the corresponding optimal control solution and infected prevalence reduction.

### Sensitivity analysis

A sensitivity analysis was performed to determine how sensitive the infected prevalence is to variations in the epidemiological parameters and in the presence of treatment. This allows us to identify which parameters play the most significant role in the disease dynamics, as well as how the prevalence sensitivity varies between baseline prevalence scenarios. For the analysis, the control function 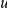 is considered a constant function of time ranging from zero to one. We then assess the variability in our prevalence at 10 years (the timescale under consideration). We do this by again utilising LHS to select 1,000 combinations of the input parameters 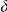, 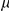, 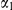, 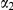 and 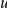. For each set of parameters, the infection rate, 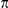, is then calculated given the untreated endemic prevalence. The value for 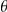 is calculated from 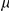 to retain a total of 1,000 IDUs. We then solve the system of ordinary differential equations using MATLAB, and track the projected prevalence at year 10 with each parameter set. We then calculate a Partial Rank Correlation Coefficient (PRCC) to assess the relative importance of each parameter in determining infected prevalence. PRCCs are widely used in sensitivity analyses in systems biology and disease transmission models to determine the importance of a parameter on a given output while fixing the other parameters at their expected value [24], [41]–[43]. The larger the absolute value of the PRCC, the more influence a parameter has on prevalence, with a PRCC magnitude greater than 0.5 and a p-value of 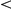
0.05 indicating the output is sensitive to changes in the input parameter.

### Discussion of parameter estimates

#### Biological parameters

We obtain the model parameters from the relevant literature on injecting drug use and HCV treatment as well as epidemiological data collected in the UK (Table 1). We present results for two common baseline chronic prevalences: 30% and 45% (approximately equivalent to 40% and 60% antibody prevalences, respectively). The exit rate is determined by the sum of the cessation of injecting rate (calculated by using the average injecting duration) and the IDU death rate. We use an average injecting duration of 11.5 years (corresponding to a cessation rate of 8.7% per year) found in [44]. This estimate for injecting duration is similar to an average estimate across England and Wales, and so is likely to be representative of many areas in the UK [45]. A recent study found an IDU mortality rate (due to overdose, suicide, and other causes) of 0.75% per year [46]. The rate of IDUs entering the population is calculated from the exit rate to retain 1,000 IDUs in the population.

**Table 1:** Biological parameter values used in the numerical simulations.

Parameter	Definition	Value	Units	Source
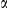	Proportion infections cured by treatment	0.625^a^	-	[11], [13]
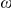	1/treatment duration	1.992^b^	per year	[11], [13]
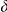	Proportion infections spontaneously clear	0.26	-	[29]
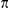	Infection rate	0.1834–0.2334	per year	Fit to 30% and 45% infection prevalences [44]
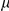	Exit rate (through death or cessation)	0.095^c^	per year	[44]–[46]
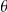	New injector entrance rate	95	per 1,000 IDUs annually	Given value to retain population of 1,000 IDUs

a Average of the genotype 1 cure rate 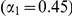 and the genotype 2/3 cure rate 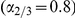.

b Exit rate calculated from the average of the genotype 1 treatment length for responders and nonresponders: 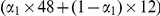 weeks and the genotype 2 treatment length, 24 weeks.

c Based on a cessation rate of 8.7% per year, and an IDU death rate of 0.75% per year.

Treatment duration and success depends on the specific genotype of HCV being treated. In general, SVR is high for IDUs with genotype 2 or 3 (75–85%), and lower for genotype 1 (40–50%) [9], [11], [13], [47]. The recommended duration of treatment for genotype 2/3 is 24 weeks for both responders or nonresponders, and for genotype 1 the duration is 48 weeks for responders and 12 weeks for nonresponders [11], [13]. In the United Kingdom, about half of the infections are genotype 1, with the remaining half 2 or 3 [11]. Hence, we take an average between the genotype 1 and 2/3 parameters for the treatment success parameter (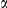), as well as the treatment duration (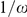). Finally, [29] performed a meta-analysis and found that 26% of infections lead to spontaneous clearance, which we use for the parameter 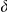. The parameter 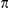 is determined for the two different prevalence scenarios by assuming that HCV amongst IDUs is in steady state, a reasonable assumption for HCV amongst IDUs the UK [44].

#### Economic parameters

A summary of the economic parameters can be found in Table 2, with the specific maximum annual budgets considered shown in Table 3. Programme treatment costs are constructed from current drug price estimates and costing studies. Depending on the type of peginterferon alfa used (2a or 3b) and body weight, the drug costs of combination peginteferon and ribvirin treatment is between 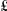
12,496–14,221 per person [11]. In addition to drug costs, we include patient evaluation, tests, screening, and consultations during and after treatment. Cost-effectiveness analyses for HCV treatment have estimated these total costs to be 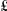
760 (in 2008/2009 GBP) for investigations of a patient who is considered for treatment, and 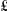
810.32–1,084.30 for consultations depending on treatment length [12]. Therefore, the total costs of delivering antiviral treatment are 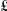
14,066–16,064 per year, and we use a mean value of 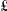
15,065 per year, varying this in the uncertainty analysis. We calculate the cost per treatment by multiplying the yearly treatment cost (which we denote 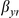) and average treatment duration (1/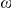).

**Table 2:** Economic parameters.

Parameter	Definition	Scenario	Value	Source
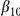	Antiviral treatment costs per treatment	all	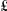 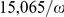	[12], [56], [73]
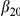	Monetarised QALY loss per treatment	A, B	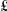 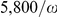	See text
		C	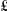 0	-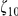	HCV infection costs per year	all	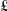 657	[12], [56], [73]
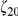	Monetarised QALY loss for HCV per year	A, B	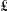 3,800	See text
		C	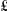 0	-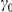	Recruitment and testing cost (per year per unit 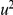 )	all	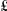 40,000	Little data, see text [53], [55]
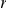	Discounting rate for costs and health utility losses (annual)	all	3.5%	[60]
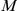	Maximum annual budget (per 1,000 IDUs)	all	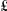 50,000–300,000	-

Scenario A: minimising health service costs and HCV health utility losses (measured in monetarised QALY loss). Scenario B: minimising health service costs and HCV health utility losses with a final time prevalence target. Scenario C: minimising only health service costs with a final time prevalence target.

**Table 3:** Net costs, net QALYs gained, and cost per QALY gained as compared to no treatment programme for the 30% and 45% HCV prevalence scenarios and various optimisation programmes.

		30% HCV prevalence			45% HCV prevalence		
Scenario	Max annual budget ( 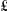 )	Net costs^1^ ( 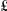 )	Net QALY gain^2^	Cost ( 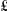 ) per QALY gained	Net costs^1^ ( 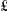 )	Net QALY gain^2^	Cost ( 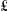 ) per QALY gained
A	50,000	−20,028	135	−148	-	-	-
	100,000	−99,210	287	−346	-	-	-
	150,000	−256,459	462	−555	327,996	293	1,120
	200,000	−520,789	668	−780	359,397	413	870
	250,000	-	-	-	332,703	550	605
	300,000	-	-	-	227,253	710	320
B	50,000	-	-	-	-	-	-
	100,000	−49,338	203	−243	-	-	-
	150,000	−46,459	216	−215	288,304	233	1,237
	200,000	−44,793	224	−200	316,510	251	1,263
	250,000	-	-	-	335,742	263	1,277
	300,000	-	-	-	349,527	272	1,285
C	50,000	-	-	-	-	-	-
	100,000	−40,348	159	−254	-	-	-
	150,000	−33,130	145	−228	213,173	179	1,192
	200,000	−23,268	137	−169	190,679	163	1,167
	250,000	-	-	-	183,950	154	1,192
	300,000	-	-	-	183,212	148	1,239

The optimal 10 year programme is determined for each scenario, and then costs and QALYs are calculated for a further 40 years (for a 50 year time horizon) in order to account for the onward prevention benefits of the treatment programme. Scenario A: minimising health service costs and HCV health utility losses (measured in monetarised QALY loss). Scenario B: minimising health service costs and HCV health utility losses with a final time prevalence target. Scenario C: minimising only health service costs with a final time prevalence target.

1 Net costs = health care costs over 50 years with the 10 year treatment programme - health care costs over 50 years with no treatment. Health care costs are defined as programme (antiviral treatment and search) costs as well as HCV infection related costs.

2 Net QALY gain = QALYs gained over 50 years with the 10 year treatment programme - QALYs gained over 50 years with no treatment.

The parameter 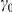 represents the cost of ensuring 100% treatment coverage in the first year, which would involve an extensive testing and recruitment programme. Programme search costs are difficult to estimate, as there is no published literature on cost analysis of IDU recruitment to services by coverage level. Systemic reviews examining costs of vaccination scale-up programmes show a lack of good methodological studies and rigorous cost analyses [48]–[50]. Nevertheless, it is reasonable to assume increasing marginal cost when attempting to increase coverage to high coverage rates, due to the assumed increased difficulty in recruitment and uptake [33]. All IDUs would need to be antibody tested. The cost of a dried blood spot antibody test is approximately 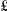
19.84, so 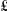
19,840 in a population of 1000 IDUs. The additional cost of RNA PCR for those who are chronically infected and will enter treatment is incorporated in the antiviral treatment costs. About one quarter of those who are antibody positive have spontaneously cleared the acute disease, and would need a PCR test to confirm their negative status. At worst, in our population of 30–45% prevalence, this amounts to about 150 IDUs per year. At a cost of 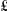
70.77 for the PCR test, the maximum cost of PCR testing is 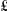
10,615.

With current dried blood spot testing technology, HCV testing can be implemented by local outreach services (such as needle and syringe programmes), however the implementation of an intensive testing programme would likely require staff and training, and potentially overheads. There is no published cost estimation for needle and syringe programmes (in particular training and staffing) in the UK [51] so estimates were taken from two other countries (Canada and Ukraine) and costs translated to the UK using the 2009 Purchasing Power Parity (PPP) Index provided by the World Bank [52]. Jacobs et al. [53] reported the yearly cost to run a local needle and syringe exchange programme (including staff, training, and overheads, but excluding syringe costs) in Edmonton, Canada as $253,553 CAD (2009). The programme distributed 565,754 needles in a year, but did not document the number of visits per year. No estimates were given for the number of needles distributed at a time, however the mean number of syringes collected per contact has been estimated at around 20 [54], leading to approximately 28,288 contacts per year, or $8.96 CAD per contact. Using the 2009 PPP conversion factor (approx. 1.76) [52] and the average exchange rate in 2009 (1.78 CAD to 1 GBP, www.x-rates.com), this results in an average cost per contact of 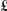
8.86 (2009). In this study, a local van was used for outreach, which is likely similar to the kind of programme which would be used in the UK if the aim was to attain high coverage. We therefore use it as the base for our programme cost estimation.

A similar analysis on data from the first year of a needle and syringe programme in Ukraine [55] includes building purchase and construction costs, giving a higher cost per contact of 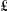
22.10 (2009). Of this amount, nearly 25% is comprised of the first year capital non-reoccurring costs. However, this is likely to be an appropriate upper bound as in subsequent years, especially in situations targeting high coverage or with low prevalence, a media campaign might be necessary. Hence, the repeated inclusion of this 25% excess cost (for capital, media, or other) seems appropriate.

In total, for 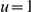 (treating at a rate of 100% of the IDU population per year), the total cost of programme outreach and testing of 1,000 IDUs would be 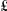
39,315, with a maximum estimate of 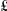
52,555. We therefore use 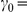 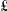 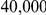 and vary this from 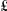
39,000 to 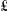
53,000 in the uncertainty analysis.

The infection costs (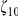) associated with a person with mild to moderate chronic HCV are approximately 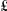
657 per year [12], [56]. These costs escalate markedly in the later stages of disease, but given the long timescale of disease progression to cirrhosis (decades) and the average injecting duration (approximately 11 years) it is assumed all infected active IDUs are either in the mild or moderate stage.

In order to quantify the quality of life reduction for active IDUs with HCV and for active IDUs with HCV undergoing treatment, we monetarise the QALY loss in each case. This approach excludes non-health benefits associated with the intervention (such as impact on productivity), but nonetheless allows us to arrive at an estimate of the net monetary benefit of the programme. In the UK, this is possible using the ‘willingness-to-pay’ threshold defined by NICE, which essentially determines the amount the UK's National Health Service is willing to pay for a treatment. The current willingness-to-pay threshold (i.e. the UK's monetarised value of a QALY) is approximately 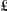
20,000 per QALY gained [57]. Estimates for the health state for a non- or ex-IDU with mild chronic HCV infection are around 0.78 QALYs per year, depending on the evaluation method [12]. Healthy active IDUs tend to have a lower baseline quality of life than non- or ex-IDUs, with estimates at around 0.85 QALYs per year [56], i.e. a 15% reduction in quality of life from the healthy non-IDU state. We use this 15% reduction to calculate the health state reduction for active IDUs with HCV, in line with other economic evaluations of HCV in active IDUs [56]. Hence, we estimate that the health state for an active IDU with HCV is 0.66 QALYs per year, i.e. an absolute reduction of 0.19 QALYs per year from the healthy active IDU state, meaning that the (monetary) benefit from successfully treating a chronically infected IDU is approximately 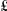
3,800 per year (0.19 QALYs/year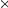 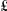
20,000/QALY). In other words, 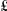
3,800 is the estimated monetary benefit foregone per year by not treating an infected IDU, hence it is essentially an opportunity cost, represented by the monetarised value of QALYs lost due to non-treatment. On the other hand, being on HCV antiviral treatment also results in a QALY reduction (from infection level) of about 0.10 QALYs per year for the duration of treatment [12], hence we assume the net reduction from the healthy active IDU state to an active IDU with HCV on treatment is approximately 0.29 QALYs per year, and therefore the cost in terms of monetarised value of QALYs lost due to treatment is approximately 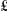
5,800 per year. These monetarised values of QALYs lost due to either infection or treatment make up the HCV health utility losses. Due to the lack of evidence surrounding utility values following treatment of those with mild (in particular, asymptomatic) HCV, we use published estimations that SVR from mild HCV results in a return to the normal health state [58], [59].

Health costs and utility losses are discounted at an annual effective rate of 3.5% per year, meaning that our cost weights decrease through time [60]. This allows for the correct cost adjustment when using a maximum implementation yearly budget. All costs are presented in UK pounds (GBP, 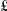) in fiscal year 2008/2009 values and updated with the Hospital and Community Health Services Pay and Prices Index [61]. We examine several budget scenarios, from a maximum yearly budget of 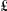
50,000 to 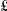
300,000 per 1,000 IDUs.

#### Parameter ranges for the uncertainty analysis

During the uncertainty analysis, each parameter is taken to be uniformly distributed, with ranges as shown in Table 4 for the biological parameters varied. For the cost parameters, the ranges for 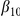, 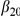, 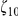, and 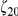 were taken to be plus and minus 10% of the values given in Table 1. Due to the high uncertainty in 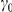, the range used was from 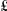
39,000 to 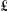
53,000.

**Table 4:** LHS sensitivity analysis on the model with constant control (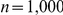).

Parameter	Range (Min, Max)	Source	PRCC^a^ 10 yr prevalence (30% baseline)	PRCC^a^ 10 yr prevalence (45% baseline)
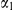	(0.40, 0.50)^b^	[11], [13], [47]	−0.6839*	−0.6904*
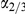	(0.75, 0.85)^b^	[9], [11], [13]	−0.7153*	−0.6762*
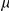	(0.05, 0.1429)^c^	[44]–[46], [74]	0.4271*	0.7234*
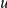	(0, 1)	-	−0.9992*	−0.9990*
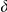	(0.22, 0.29)	[29]	−0.0387	−0.0338

a Partial Rank Correlation Coefficient,

b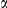 is calculated as an average of the genotype treatment success rates, 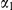 and 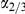.

c The exit rate is calculated from the range of the genotype 1 treatment length for responders and nonresponders: 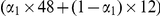 weeks and the genotype 2 length: 24 weeks.

*denotes a p-value of below 0.05.

## Results

If only health service costs are minimised (with no requirement to reduce health utility losses or reach a final time prevalence reduction), the optimal solution is to treat no one. In each of the scenarios considered below, we add further considerations to this baseline case.

### Scenario A: Minimising health service costs and HCV health utility losses (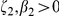)

This scenario represents an ‘ideal’ situation, where policymakers are motivated to minimise health service costs and health utility losses associated with HCV, limited only by budget restrictions. The optimal programme is to spend the maximum possible amount each year on the treatment programme, which succeeds in reducing infections and QALY losses.

**Figure 1:**
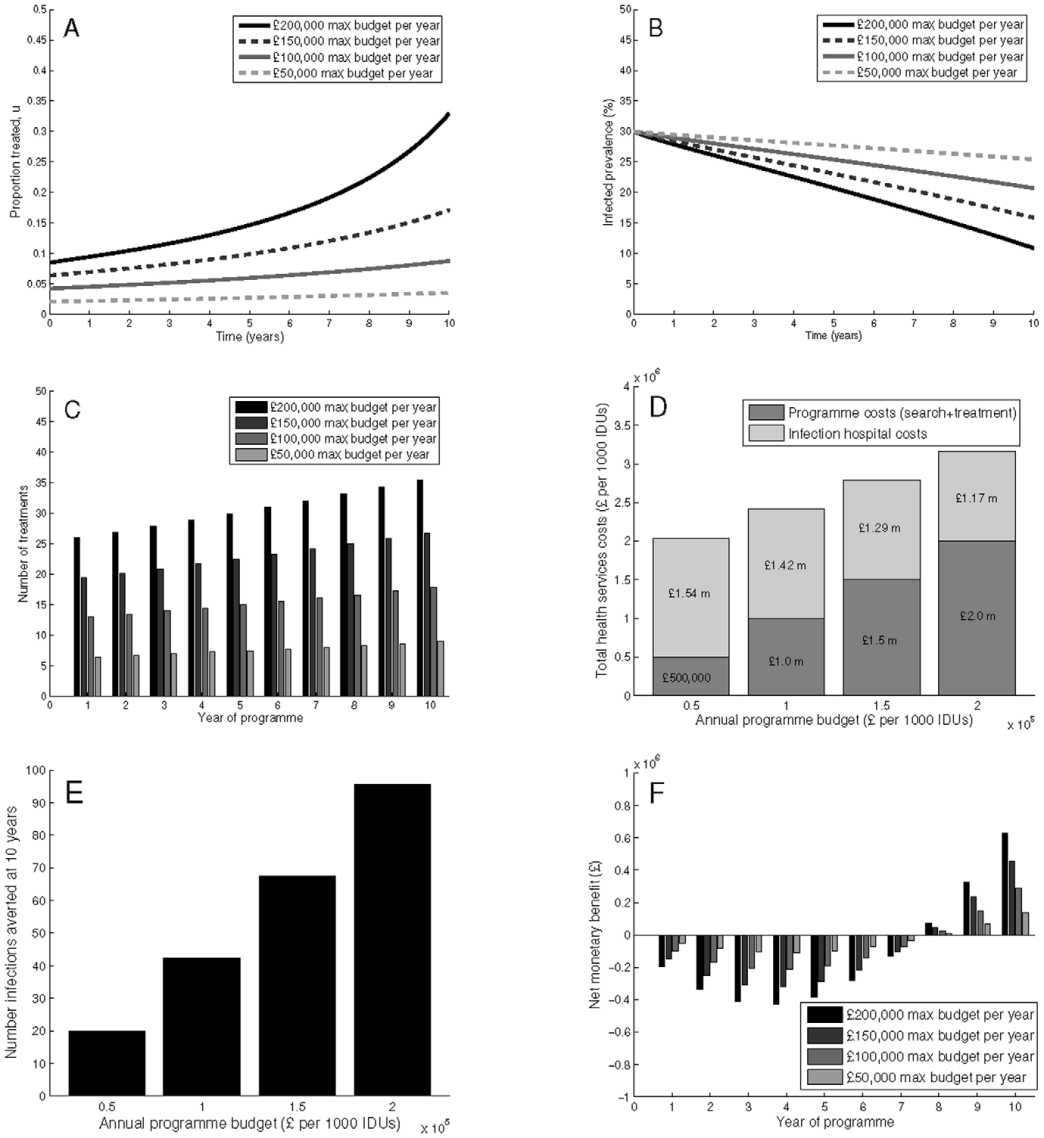
Scenario A: Minimising health service costs and HCV health utility losses. Simulations are with a 30% baseline prevalence, showing (A) programme coverage, (B) prevalence reductions, (C) number of treatments, (D) total health service costs (comprised of programme costs and infection costs), (E) infections averted, and (F) net monetary benefit. Parameters used are as shown in Tables 1–2, with 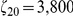, 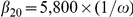, 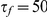, and with no final time prevalence constraint.

If the objective is to minimise health service costs and health utility loss related to HCV infection, Figure 1 shows the potential impact of the optimal treatment programmes for various maximum yearly programme budgets with a 30% baseline prevalence. In all budget scenarios, the optimal number treated increases over time, due to the discounting treatment costs and subsequent ability to increase treatment allocation. Figure 1 shows that depending on the maximum annual budget (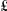
50,000 to 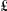
200,000 per 1,000 IDUs annually), the number of treatments allocated yearly varies from 7 to 37. With a 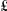
100,000 maximum annual programme budget, the prevalence decreases from 30% to about 21% at year 10 with a total of 43 cases (per 1,000 IDUs) averted. This equates to a total programme spend per infection averted of 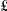
23,597. The infection related costs reach 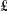
1.42 million by 10 years. Net monetary benefit at 10 years is 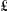
291,088. The cost per QALY gained with a 50 year time horizon is −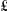
346, with negative net costs and positive net QALYs gained as compared to no treatment, indicating that the programme is cost saving (Table 3).

When the budget constraint is increased to 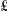
200,000 per year, the prevalence decreases to below 12% within a decade. Additionally, 96 infections are averted by 10 years, resulting in a reduction in cost per infection averted to 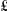
20,945. As compared to the lower budget scenario, the increased programme costs are partly offset by reduced infection-related costs of 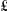
1.17 million at 10 years. Net monetary benefit increases to 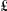
630,111. Additionally, increasing the budget saves more in terms of cost over a 50 year time horizon and gains more QALYs, resulting in a cost per QALY gained of −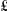
780, indicating that this programme is more cost-effective than one with a lower budget (Table 3). Results are qualitatively similar for the 45% baseline prevalence scenarios, however with the same yearly budget the relative reduction in prevalence is smaller, there are fewer infections averted, and the cost per averted infection is higher (Supporting Information Figure S1). Furthermore, the programme results in fewer QALY gains, and the cost per QALY gained is higher, at 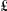
870 (Table 3).

Overall, increasing the yearly budget results in greater short-term reductions in prevalence, increased infections averted, lower programme cost per infection averted, and substantial reductions in infection related costs at year 10 due to the subsequent prevention effect. For a given yearly budget, the impact is higher in lower prevalence areas.

The optimal programme strategy and results presented above are identical if the objective is instead to minimise prevalence at the end of year 10 while being constrained by the same budget restrictions. In other words, the programme which minimises prevalence at the end of year 10 is the one where the entire budget is spent every year, and this strategy also has the lowest cost per QALY gained.

### Scenario B: Minimising health service costs and HCV health utility losses with a final time prevalence target (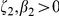, 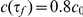)

In a less ‘ideal’ scenario, policymakers could be motivated by a political constraint as well, and specify a need to reduce prevalence by a specific amount within 10 years. Therefore, in this case we examine the optimal timing and intensity of a programme where the desire is to achieve a specific prevalence reduction by the final time, while also minimising total health service costs and HCV health utility losses. These objectives are again constrained by yearly budget restrictions. In these simulations, the final time prevalence is specified as a necessary condition, such that the prevalence at 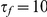 years is reduced by a relative 20% (so, from 30% to 24% or 45% to 36%).

**Figure 2:**
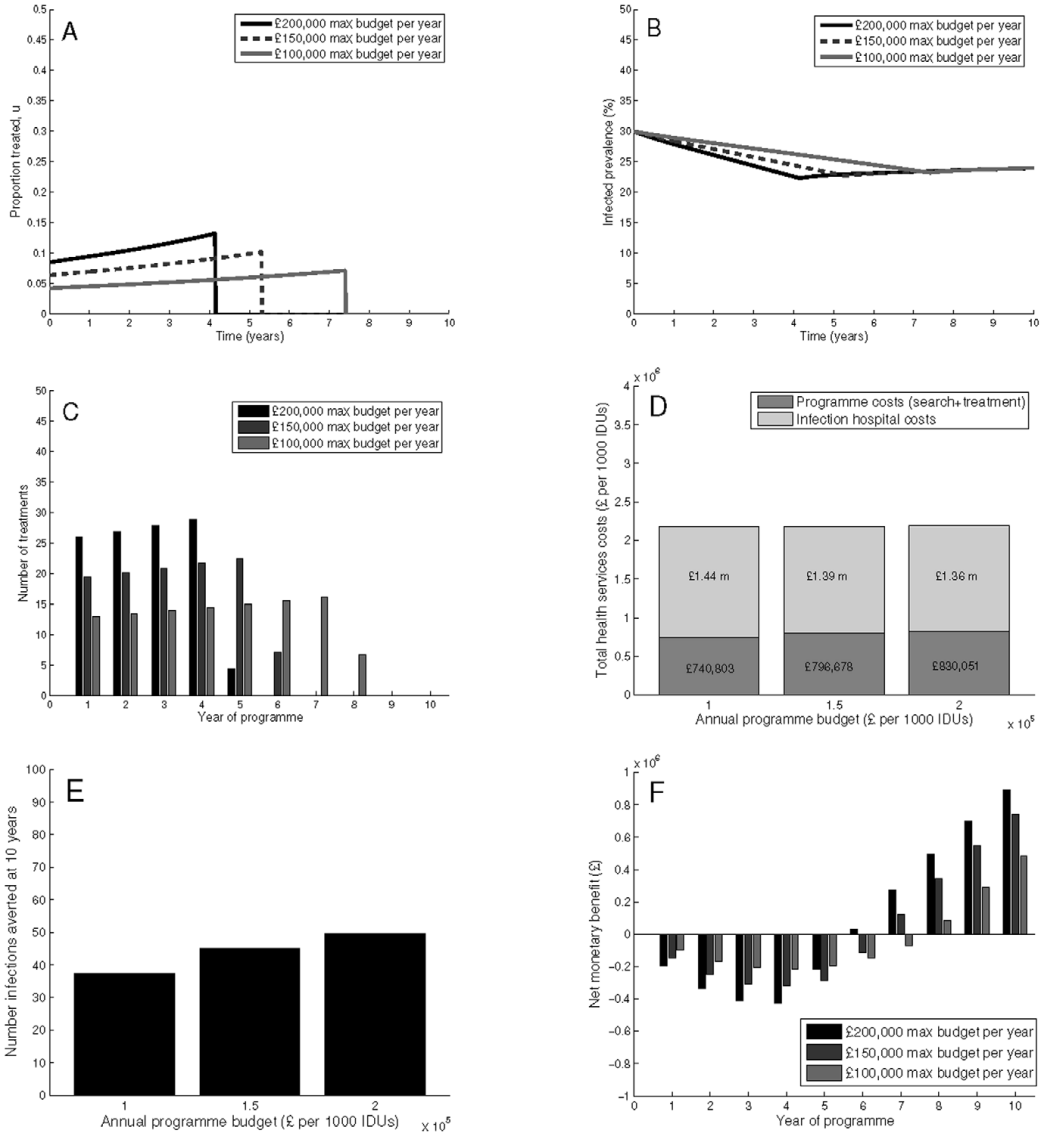
Scenario B: Minimising health service costs and HCV health utility losses with a final time prevalence target. Simulations are with a 30% baseline prevalence, showing (A) programme coverage, (B) prevalence reductions, (C) number of treatments, (D) total health service costs (comprised of programme costs and infection costs), (E) infections averted, and (F) net monetary benefit. Parameters used are as shown in Tables 1–2, with 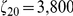, 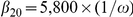, and a final time prevalence constraint.

Numerical solutions indicate that the best strategy with a final time target including costs related to health state reductions is an initial, intense programme (Figure 2). In the 30% baseline prevalence scenario, the 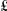
50,000 maximum budget scenario is not sufficient to result in a 20% relative reduction in prevalence. With an annual budget of 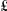
100,000, the optimum is an 8 year programme of increasing treatment coverage (expanding from 4% to 8% of infected IDUs), treating 13–17 people per year in the first seven years where the full budget is spent, and 7 people in the eighth year (Figure 2). This programme results in an initial swift decrease in prevalence, slightly overshooting the 10 year target prevalence in year 8, and eventually rebounding to the target by the end of year 10. The programme averts 37 infections resulting in a programme cost per infection averted of 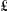
19,888. The total costs of the programme (treatment and search) reaches just over 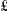
740,803 with the infection related costs reaching 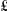
1.44 million. In this scenario, net costs as compared to no treatment are still negative, but fewer QALYs are averted as compared to Scenario A, and cost per QALY gained is higher at −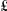
243, however the programme is still cost-saving (Table 3).

Increasing the budget to 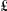
200,000 decreases the duration of the optimal programme to five years, with a lower level of treatments (26–30) for the first four years, tailing off with 5 treatments in the fifth year. This strategy increases the programme cost to 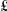
830,051, but averts more infections (50 by year 10), resulting in a lower programme cost per infection averted of 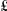
16,737. Furthermore, the infection related costs are reduced to 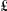
1.36 million. However, the cost per QALY gained is slightly higher than in the lower budget scenario, at −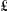
200 due to the higher programme cost (Table 3).

With a 45% baseline prevalence scenario, the optimal programme is still an initial programme, but the programme duration to reduce prevalence by 20% is longer (Supporting Information Figure S2). With an annual budget of 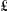
200,000, the programme spans 7 years instead of the 5 years in the lower prevalence scenario. Total programme and infection costs are substantially higher, at 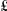
1.35 million and 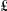
2.1 million, respectively. Additionally, fewer infections are averted (40 in 10 years), and the cost per infection averted is nearly double that of the 30% prevalence scenario. Similarly, the cost per QALY increases to 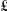
1,263 (Table 3).

### Scenario C: Final time prevalence reduction only (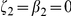, 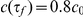)

**Figure 3:**
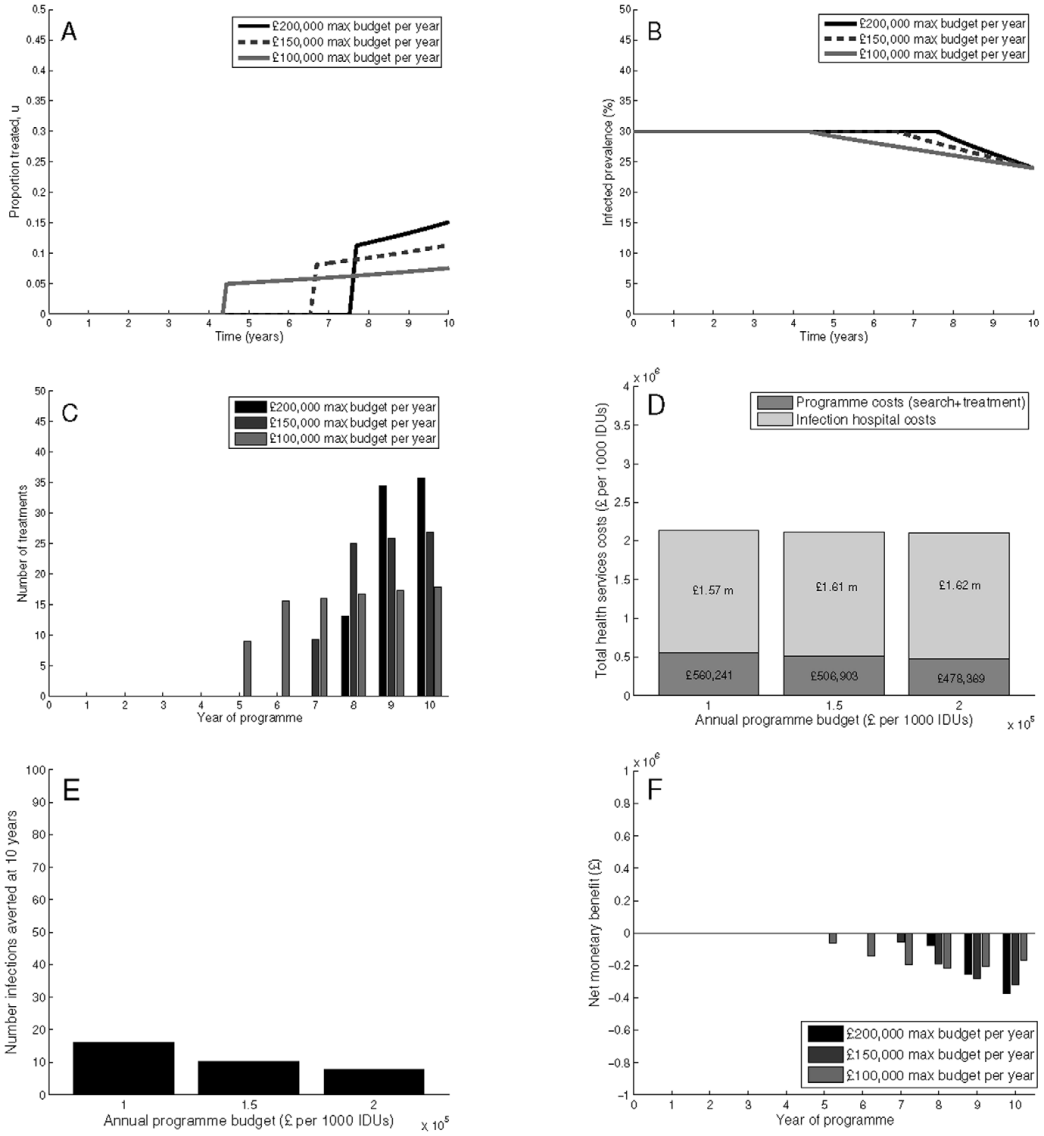
Scenario C: Minimising only health service costs with a final time prevalence target. Simulations are with a 30% baseline prevalence, showing (A) programme coverage, (B) prevalence reductions, (C) number of treatments, (D) total health service costs (comprised of programme costs and infection costs), (E) infections averted, and (F) net monetary benefit. Here, we neglect health utility losses. Parameters used are as shown in Tables 1–2, with 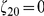, 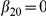, and a final time prevalence target constraint.

In the least ‘ideal’, but perhaps most relevant and ‘real-world’ scenario, policymakers may be motivated solely by a political commitment to reduce prevalence by a specific amount, neglecting the loss of health utilities associated with infection. As compared to Scenario B, where there is a bias towards early and intensive treatment to reduce the cumulative number of infections, neglecting health utility losses results in very different optimal programmes (Figure 3). In the 30% prevalence scenario, with a maximum budget at 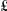
200,000 per year, the optimal programme is implemented in the final three years only, increasing treatment coverage from 11% to 16%. This results in treating 13–36 people per year, costing the programme only 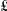
478,369, but infection costs reach 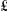
1.62 million. Since the costs associated with infections are much lower when neglecting loss of health utility, the optimal strategy shifts toward achieving the target prevalence with fewer treatments, whereas with health utility losses included in the objective, the optimal strategy seeks to decrease a greater number of the infections by treating earlier instead of later. Hence, the number of infections averted is only 8 per 1,000 IDUs at year 10, and the cost per infection averted is substantially increased to over 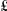
62,324. Importantly, in this scenario, the programme does not result in a net monetary benefit at 10 years, with costs exceeding benefits throughout the decade. The cost per QALY gained (−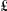
169) is slightly higher than Scenarios A and B, with fewer QALYs gained, and higher costs (Table 3).

Decreasing the yearly budget to 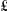
100,000 results in a longer programme, lasting years 5 through 10, increasing treatment coverage from 5% to 8%. Reducing the budget increases programme costs (to 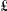
560,241), but decreases infection costs to 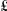
1.57 million. Furthermore, decreasing the maximum annual budget increases the treatment programme duration, resulting in more infections averted (nearly 16 per 1,000 IDUs by year 10). Additionally, the cost per infection averted is substantially less, at just over 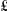
35,087. This indicates that although a higher annual budget can achieve the same prevalence reduction with a shorter programme duration, achieving earlier prevalence reductions (for example, by treating fewer but initiating the programme earlier) results in more infections averted and a reduced cost per infection averted. The qualitative shape of the programme (delayed until final years) is unchanged if discounting is neglected.

For the 45% prevalence scenario, the optimum is also a late initiated programme, but with a longer programme duration to achieve the target prevalence (Supporting Information Figure S3). The programmes begin in years 4–8 and escalate until the final year. Cumulative programme costs are roughly equal (at the 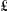
200,000 annual budget) and 15% higher (at the 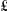
150,000 annual budget) than in the 30% prevalence scenario, and the cost per QALY gained is higher than in scenarios A and B, or for any of the low prevalence scenarios, at about 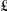
1,200.

### Uncertainty analysis of optimal control solution

**Figure 4:**
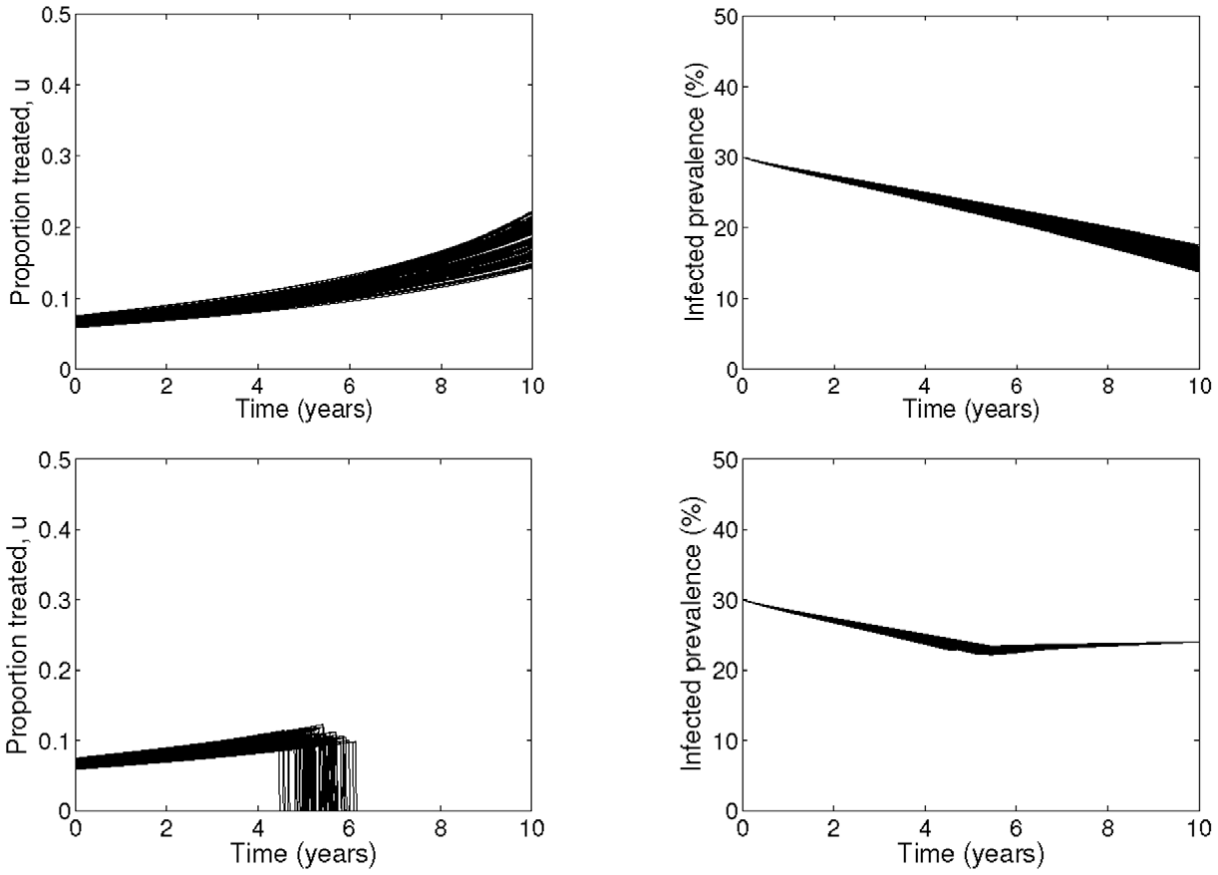
Uncertainty analysis results for optimal control. Simulations are shown for Scenario A (top) and Scenario B (bottom). The baseline prevalence is 30%, and maximum yearly budget is limited to M = 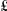
150,000. The cost coefficients 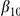, 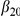, 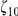, 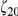 are uniformly distributed with means given in Table 1 and the ranges given by plus and minus 10% of the means. The range for 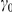 is 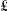
39,000–53,000. The parameters 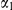, 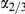, 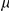 and 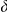 are uniformly distributed with the ranges given in Table 4.

The impact of uncertainty of our parameters on the optimal control and prevalence reductions for various maximum budget scenarios are shown in Figure 4 for 30% baseline prevalence and maximum yearly budget of 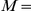 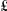
150,000 with and without the final time prevalence target (including monetarisation of QALYs). The results for 45% baseline prevalence with a maximum annual budget of 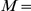 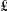
200,000 are shown in Figure S4 of the Supporting Information. Despite the uncertainty in both the biological and economic cost parameters, the qualitative results remain unchanged, with only small variations of at most two years in the duration of treatment programme.

### Sensitivity analysis

The results of the sensitivity analysis are found in Table 4, which shows how sensitive the 10 year prevalence is to changes in the epidemiological parameters. Here, we assume the treatment rate is constant through time. This allows for the assessment of which parameters to which the prevalence is most sensitive. In both prevalence scenarios, the endemic prevalence is most sensitive to the treatment rate, which indicates that treatment could play an important role in reducing prevalence. The 45% prevalence scenario is then most sensitive to the exit rate (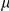), followed by the treatment cure rates (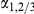). By contrast, the 30% prevalence scenario is more sensitive to the treatment cure rates than exit rate. This indicates that at higher prevalences, variations in injecting duration between sites can significantly alter impact projections; at lower prevalences variation in injecting duration would have less of an impact on prevalence at 10 years. As the sensitivity coefficient of the exit rate is positive, increasing the injecting duration would lead to an increase in prevalence. Conversely, increasing treatment cure rates lead to decreasing prevalence. The sensitivity results indicate that in high prevalence scenarios, aside from antiviral treatment, initiatives reducing injecting duration would have the most effect on reducing prevalence. Notably, in both prevalence scenarios the 10 year prevalence is not sensitive to the variation in the spontaneous clearance rate. Hence, for example, a 10% change in spontaneous clearance would have substantially less of an impact on prevalence than the same percent change in injecting duration.

## Discussion

### Main Findings

We use optimal control theory to determine the optimal timing and intensity of an HCV antiviral treatment programme for active IDUs with a variety of policy objectives, budget constraints, and prevalence settings. The aim is to aid in the design and implementation of treatment programmes aimed at targeting active IDUs and utilising antiviral treatment as a prevention strategy. From a public health and economic perspective, if there is a fixed yearly budget then the ideal strategy is an immediate programme of maximum intensity, with the maximum budget constraint spent each year on treatment. This minimises health service costs and HCV health utility losses. This results in high health service costs, but high numbers of infections averted (up to 90 per 1,000 IDUs for the budgets considered) and substantial (up to 60%) reductions in prevalence at 10 years, depending on annual programme budget and prevalence. At an HCV chronic prevalence of 30%, the 10 year programme is cost-saving over a 50 year time horizon, and has the lowest cost per QALY gained as compared to other scenarios due to the substantial prevention benefit. For the same annual budget, a higher baseline prevalence results in higher costs per QALY gained, and the programme is no longer cost saving (though well below the willingness-to-pay threshold), as the prevention impact is less. Increasing the annual programme budget results in greater short-term (10 years) reductions in prevalence, reduced cost per infection averted, substantial reductions in infection-related costs, and greater cost-effectiveness (lower costs per QALY gained) due to the subsequent prevention effect. Since the costs per QALY gained are below the current willingness-to-pay thresholds for the UK and elsewhere (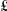
20,000–30,000 per QALY gained), these results suggest that increasing the budget allocated to HCV treatment amongst IDUs would be an improved strategy.

A programme may have a policy objective of reducing HCV prevalence by a certain amount over 10 years. In this case, the optimal programme implementation strategy changes substantially depending on whether or not there is a further objective of minimising the health impact of the disease (measured by monetarised QALY loss). If the policymaker desires to minimise the loss of health utility related to HCV infection in addition to health service costs, the optimal strategy is to gradually increase coverage over the first part of the decade and then stop after the desired prevalence is reached. This immediate, but shorter programme reduces the cost of the treatment programme, but increases HCV infection hospital costs and averts fewer infections. Therefore, although the programme achieves its policy objective (to reduce prevalence by 20%), it fails to have the full prevention impact and is less cost-effective (though still well below the cost-effectiveness willingness-to-pay threshold) than the previous scenario which did not have a prevalence target. With this programme, increasing the annual budget results in more infections averted, but a higher cost per QALY gained due to the slightly higher programme costs.

However, where policymakers are motivated only by achieving the target prevalence reduction with minimum health service costs (and do not consider HCV health utility losses) then the optimal timing of scale-up changes substantially. In this case, the ‘optimal’ programme is to delay initiating the treatment programme until the final few years, resulting in the desired prevalence reduction but with lower programme costs. However, this delayed programme results in many fewer infections averted, higher costs per infection averted, and higher HCV infection-related costs. This indicates that implementers who neglect the loss of health utilities associated with being HCV infected but only consider health service costs may plan the programme in such a way that it reduces its cost-effectiveness. The ‘optimal’ programme includes treatment only because of the required prevalence reductions. Moreover, in this scenario, increasing the maximum budget reduces programme duration and decreases health services costs but results in fewer infections averted, and increased cost per infection averted. Hence, in this scenario a lower budget programme results in greater impact and programme cost-effectiveness. Finally, all our scenarios show, with a fixed annual budget, greater impact (measured by infections averted, or prevalence reductions) and cost-effectiveness will be achieved in lower prevalence areas.

### Strengths and limitations

The use of optimal control theory in public health programme delivery and design is still relatively unexplored. As such, there are a number of methodological issues mentioned in this paper which need to be addressed before real-world implementation of this technique. First, we assume a quadratic search cost function, to reflect the increasing unit costs related to recruitment and testing with higher programme coverage. Although studies have shown that in many cases these unit costs are not linear [62]–[65], the precise shape of this curve (be it quadratic, cubic, or other) is still unclear, and likely varies between situations. We assume that costs for a given coverage level are equal for different baseline prevalence scenarios, although it is possible that search costs for a given treatment coverage could be lower in different settings. Further research into the most accurate form of this cost function, as well as how it varies in real-world settings, would strengthen the confidence in the specific predictions of the model.

Second, the actual costs associated with the scale-up of treatment coverage are difficult to estimate, and likely vary considerably depending on target population and current coverage level. Most traditional cost analyses neglect the additional costs related to increased coverage and recruitment, but these costs can often be significant. Proper quantification of costs related to media campaigns, outreach networks, and testing coverage would strengthen this analysis. In particular, it is likely that the costs of increasing coverage vary depending on baseline coverage level, target population, and population size. Additional studies quantifying costs related to identifying a greater proportion of the hidden IDU community, and expanded testing and treatment recruitment would aid in parameterising future economic models.

Third, given the lack of clinical studies examining the potential effect of antiviral treatment to prevent transmission of HCV amongst active IDUs, our study is based on a previously developed mathematical model. Therefore, the findings are based on model projections of the treatment effect and not experimental evidence. Furthermore, the underlying disease transmission model neglects heterogeneity within the injecting drug user population, which may alter the efficacy of a treatment programme and the quantitative projections presented here. For example, shifts in genotype distribution may serve to increase/decrease programme efficacy. Additionally, it is highly likely there will be heterogeneities in treatment presentation, completion, and post-treatment behaviour and risk, among individual IDUs and also at different times during an individual's injecting career. Unfortunately, there is insufficient evidence to parameterise this heterogeneity, which can only be incorporated once additional clinical evidence has been collected.

Fourth, it is also important to note that we assume a constant population of active IDUs, which is an appropriate approximation in the UK, but may not apply to settings such as the Netherlands, which has a shrinking IDU population due to a reduction in incidence of new injectors. However, many countries do not have a declining population, primarily because given the prolonged duration of injecting, changes in incidence take a long time to be observed in changes in prevalence [66]. Nevertheless, further studies could explore the impact of relaxing this assumption and apply the model to areas with a nonconstant population size.

Fifth, the cost per QALY gained estimates have several limitations. Most notably, the model does not track former IDUs, and therefore underestimates the QALYs gained after cessation of drug use by those who are treated or prevented from infection while active IDUs. A detailed economic evaluation aimed at evaluating the cost-effectiveness of treating active IDUs would therefore include subsequent HCV disease states often reached after cessation of drug use (cirrhosis, hepatocellular carcinoma, liver transplant, etc.) and calculate detailed costs and QALYs for each state. There is also considerable uncertainty surrounding QALY fractions for active IDUs (either uninfected or HCV infected), and a full economic evaluation of treatment in active IDUs would need to address this uncertainty, which we have neglected.

Finally, we neglect any additional health costs due to non-HCV related illness resulting from increased lifespan from successful HCV antiviral treatment. Previous economic evaluations of HCV antiviral treatment have neglected additional costs or QALY losses due to non-HCV related diseases which may occur due to increasing lifespan, and we make a similar assumption [12]. However, if these costs and QALYs could be quantified, the inclusion would provide a fuller picture of cost-effectiveness. Furthermore, due to the focus on current IDUs only, we neglect costs and utilities associated with HCV progression past the mild or moderate state. We believe this is an appropriate first assumption, as any programme targeting IDUs who are actively injecting is likely to treat the disease at the mild stage which would result in minimal future HCV-related health service costs. However, it is possible that an increase in life-expectancy could accrue additional costs, particularly if broader societal costs (such as those related to injecting drug use) are included. Unfortunately, the lack of data relating to how antiviral treatment of IDUs alters injecting behavior and subsequent societal costs makes this difficult to include in an analysis. Future work quantifying the cost and impact of treatment on IDU behaviour would enable a more detailed study than the work presented here.

### Evidence from other studies

There is an established body of literature describing the use of optimal control theory on biological systems [67]–[72], with some applications towards control measures for infectious diseases [24]–[27]. To our knowledge, this is the first application of this technique to the control of HCV amongst injecting drug users. The use of antiviral treatment as prevention of HCV amongst IDUs has been proposed by several modelling studies [16]–[19], but the experimental or clinical data are limited and probably subject to considerable selection bias. Nevertheless, the available evidence suggests that IDUs can be treated successfully, indicating the potential for using antiviral treatment as a control measure.

### Implications and future work

Despite the application of a number of prevention measures, HCV remains an important public health concern in the IDU population. Antiviral treatment for HCV has been established as effective and cost-effective, but uptake of therapy remains low among active IDUs, and is rarely encouraged. Our previous modelling work has shown that antiviral therapy could play a valuable role in controlling the HCV epidemic amongst IDUs. Despite this, treatment rates among IDUs remain low (less than 1%) even in countries such as the UK and Australia where treatment is recommended under a national health care system. Recently, several action plans have been developed which aim to allocate specific resources to initiating HCV treatment programmes [20]–[22], though none specifically aimed at active IDUs and for the expressed purpose of prevention. There is increasing interest in developing treatment programmes aimed at treating active IDUs for prevention [23], and as such we aimed to determine the optimal timing and intensity of an HCV antiviral treatment programme for active IDUs, given various resource, policy, and prevalence constraints and objectives as could be seen in the real world. We find that incorporating different budget constraints and policy objectives plays an important role in determining the optimal programme structure, and subsequent programme cost-effectiveness (measured by cost per QALY gained). Extensions of our current model will explore the optimal allocation of the currently available prevention options (such as needle and syringe programmes and opiate substitution therapy) given resource constraints, in order to best combat the spread of HCV amongst injecting drug users. This could inform policymakers on which interventions to spend money first, if alternating interventions is the best strategy, or how specific combinations of interventions at different stages could best combat the disease.

Though used widely in other disciplines, the use of optimal control theory with reference to public health programme implementation and resource allocation is fairly limited. Most optimal resource allocation models only focus on optimal allocation at a single timepoint, while optimal control theory allows for the optimal allocation to change continuously over time. We believe this powerful technique could play a key role in guiding policy decisions and programme design, especially in limited resource scenarios. Future research in this field should focus on properly quantifying costs related to increased programme coverage and scale-up, which are currently difficult to estimate.

## Supporting Information

Figure S1
**Scenario A: Minimising health service costs and HCV health utility losses.** Simulations are with a 45% baseline prevalence, showing (A) programme coverage, (B) prevalence reductions, (C) number of treatments, (D) total health service costs (comprised of programme costs and infection costs), (E) infections averted, and (F) net monetary benefit. Parameters used are as shown in Tables 1–2, with 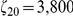, 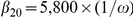, 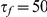, and with no final time prevalence target constraint.(TIFF)

Figure S2
**Scenario B: Minimising health service costs and HCV health utility losses with a final time prevalence target.** Simulations are with a 45% baseline prevalence, showing (A) programme coverage, (B) prevalence reductions, (C) number of treatments, (D) total health service costs (comprised of programme costs and infection costs), (E) infections averted, and (F) net monetary benefit. Parameters used are as shown in Tables 1–2, with 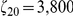, 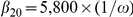, and a final time prevalence target constraint.(TIFF)

Figure S3
**Scenario C: Minimising only health service costs with a final time prevalence target.** Simulations are with a 45% baseline prevalence, showing (A) programme coverage, (B) prevalence reductions, (C) number of treatments, (D) total health service costs (comprised of programme costs and infection costs), (E) infections averted, and (F) net monetary benefit. Here, we neglect the health utility losses. Parameters used are as shown in Tables 1–2, with 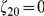, 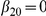, and a final time prevalence target constraint.(TIFF)

Figure S4
**Uncertainty analysis results for optimal control.** Simulations are shown for Scenario A (top) and Scenario B (bottom). The baseline prevalence is 45%, and maximum yearly budget is limited to M = 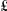
200,000. The cost coefficients 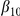, 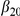, 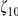, 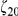 are uniformly distributed with means given in Table 2 and the ranges given by plus and minus 10% of the means. The range for 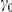 is 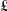
39,000–53,000. The parameters 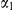, 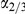, 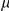 and 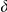 are uniformly distributed with the ranges given in Table 3.(TIFF)

## References

[1] Alberti A, Benvegnu L (2003). Management of hepatitis C.. Journal of Hepatology.

[2] Shepard CW, Finelli L, Alter MJ (2005). Global epidemiology of hepatitis C virus infection.. The Lancet Infectious Diseases.

[3] Seeff LB (2009). The history of the “natural history” of hepatitis C (1968–2009).. Liver International.

[4] ACMD (2009). The primary prevention of hepatitis C among injecting drug users..

[5] Page-Shafer K, Pappalardo BL, Tobler LH, Phelphs BH, Edlin BR (2008). Testing strategy to identify cases of acute hepatitis C virus (HCV) infection and to project HCV incidence rates.. Journal of Clinical Microbiology.

[6] Judd A, Hickman M, Jones S, McDonald T, Parry JV (2005). Incidence of hepatitis C virus and HIV among new injecting drug users in London: prospective cohort study.. British Medical Journal.

[7] Hahn JA, Page-Shafer K, Lum PJ, Bourgois P, Stein E (2002). Hepatitis C virus seroconversion among young injection drug users: Relationships and risks.. The Journal of Infectious Diseases.

[8] Sweeting M, Hope V, Hickman M, Ncube F, Ramsay M (2009). Hepatitis C infection among injecting drug users in England and Wales 1992–2006: there and back again?. American Journal Epidemiology.

[9] Hoofnagle J, Seeff L (2006). Peginterferon and ribavirin therapy for chronic hepatitis C.. New England Journal of Medicine.

[10] NICE (2000). Inteferon alfa (pegylated and non-pegylated) and riabirin for the treatment of chronic hepatitis C..

[11] NICE (2006). Peginterferon alfa and ribavirin for the treatment of mild chronic hepatitis C..

[12] Shepherd J, Jones J, Hartwell D, Davidson P, Price A (2007). Interferon alfa (pegylated and non-pegylated) and ribavirin for the treatment of mild chronic hepatitis C: a systematic review and economic evaulation.. Health Technology Assessment.

[13] NIH (2002). Management of hepatitis C. 2002..

[14] Grebely J, Conway B, Raffa J, Lai C, Krajden M (2006). Uptake of hepatitis C virus (HCV) treatment among injection drug users (IDUS) in Vancouver, Canada.. Journal of Hepatology.

[15] Seal K, Kral A, Lorvick J, Gee L, Tsui J (2005). Among injection drug users, interest is high, but access low to HCV antiviral therapy.. Journal General Internal Medicine.

[16] Martin NK, Vickerman P, Foster GR, Hutchinson SJ, Goldberg DJ (2011). Can antiviral therapy for hepatitis C reduce the prevalence of HCV among injecting drug user populations? A modelling analysis of its prevention utility.. Journal of Hepatology.

[17] Martin NK, Vickerman P, Hickman M (2011). Mathematical modelling of hepatitis C treatment for injecting drug users.. Journal of Theoretical Biology.

[18] Zeiler I, Langlands T, Murray J, Ritter A (2010). Optimal targeting of hepatitis C virus treatment among injecting drug users to those not enrolled in methadone maintenance programs.. Drug and Alcohol Dependence.

[19] Vickerman P, Martin N, Hickman M (2010). Can hepatitis C virus treatment be used as a prevention strategy? Additional model projections for Australia and elsewhere?. Drug and Alcohol Dependence.

[20] Australian Government, Department of Health and Aging (2010). Third National Hepatitis C Strategy 2010–2013..

[21] The Scottish Government (2008). Hepatitis C Action Plan for Scotland: Phase II: May 2008–March 2011..

[22] Public Health Wales (2008). Blood Bourne Viral Hepatitis Action Plan for Wales: 2010–2015..

[23] The London Joint Working Group for Substance Misuse and Hepatitis C (2011). Tackling the problem of hepatitis C, substance misuse and health inequalities: delivering a consensus for London..

[24] Miller-Neilan R, Schaefer E, Gaff H, Fister KR, Lenhart S (2010). Modeling optimal intervention strategies for cholera.. Bulletin of Mathematical Biology.

[25] Jung E, Lenhart S, Feng Z (2002). Optimal control of treatments in a two-strain tuberculosis model.. Discrete and Continuous Dynamical Systems Series B.

[26] Caetano M, Yoneyama T (2001). Optimal and sub-optimal control in dengue epidemics.. Optimal Control Applications and Methods.

[27] Ambruster B, Brandeau M (2010). Cost-effiective control of chronic viral diseases: Finding the optimal level of screening and contact.. Mathematical Biosciences.

[28] ECMDDA (2004). Hepatitis C and injecting drug use: Impact, costs and policy options..

[29] Micallef J, Kaldor JM, Dore GJ (2006). Spontaneous viral clearance following hepatitis C infection: a systematic review of longitudinal studies.. Journal of Viral Hepatitis.

[30] Neumann AU, Lam NP, Dahari H, Gretch DR, Wiley TE (1998). hepatitis C viral dynamics in vivo and the antiviral efficacy of interferon- therapy.. Science.

[31] Litwin AH, Harris KA, Nahvi S, Zamor PJ, Soloway IJ (2009). Successful treatment of chronic hepatitis C with pegylated interferon in combination with ribavirin in a methadone maintenance treatment program.. Journal of Substance Abuse Treatment.

[32] Chack E, Saab S (2010). Pegylated interferon and ribavirin dosing strategies to enhance sustained virological response.. Current Hepatitis Reports.

[33] Noah N (1983). The strategy of immunization.. Journal of Public Health.

[34] Pontryagin LS, Boltyanskii VG, Gamkrelize RV, Mishchenko EF (1962). The Mathematical Theory of Optimal Processes.

[35] Fleming WH, Rishel RW (1975). Deterministic and Stochastic Optimal Control.

[36] Bryson AE, Ho YC (1969). Applied Optimal Control.

[37] Lenhart S, Workman JT (2007). Optimal Control Applied to Biological Models. Mathematical and Computational Biology Series.

[38] Kopp RE, Moyer HG (1965). Necessary conditions for singular extremals.. AIAA Journal.

[39] McDanell JP, Powers WF (1971). Necessary conditions for joining optimal singular and nonsingular subarcs.. SIAM Journal on Control and Optimization.

[40] Vincent TL, Grantham WJ (1997). Nonlinear and Optimal Control Systems.

[41] Blower S, Dowlatabadi H (1994). Sensitivity and uncertainty analysis of complex models of disease transmission: an HIV model, as an example.. International Statistical Review.

[42] Marino S, Hogue I, Ray C, Kirschner D (2008). A methodology for performing global uncertainty and sensitivity analysis in systems biology.. Journal of Theoretical Biology.

[43] Blower SM, Gershengorn HB, Grant RM (2000). A tale of two futures: HIV and antiretroviral therapy in San Francisco.. Science.

[44] Hickman M, Hope V, Brady T, Madden P, Jones S (2007). Hepatitis C virus (HCV) prevalence, and injecting risk behaviour in multiple sites in England in 2004.. Journal of Viral Hepatitis.

[45] Sweeting MJ, De Angelis D, Ades AE, Hickman M (2009). Estimating the prevalence of ex-injecting drug use in the population.. Statistical Methods in Medical Research.

[46] Hickman M, Hope V, Coleman B, Parry J, Telfer M (2009). Assessing IDU prevalence and health consequences (HCV, overdose and drug-related mortality) in a primary care trust: Implications for public health action.. Journal of Public Health (Oxf).

[47] Hadziyannis S, Sette H, Morgan T, Balan V, Diago M (2004). Peginterferon-alpha2a and ribavirin combination therapy in chronic hepatitis C: a randomized study of treatment duration and ribavirin dose.. Annals of Internal Medicine.

[48] Pegurri E, Fox-Rushby J, Damian W (2005). The effects and costs of expanding the coverage of immunisation services in developing countries: A systematic literature review.. Vaccine.

[49] Batt K, Fox-Rushby J, Castillo-Riquelme M (2004). The costs, effects and cost-effectiveness of strategies to increase coverage of routine immunizations in low-and middle-income countries: systematic review of the grey literature.. Bulletin of the World Health Organization.

[50] Corluka A, Walker D, Lewin S, Glenton C, Scheel I (2009). Are vaccination programmes delivered by lay health workers cost-effective? A systematic review.. Human Resources for Health.

[51] NICE (2009). Costing statement: Needle and syringe programmes..

[52] World Bank (2009). Gross domestic product PPP.. http://siteresources.worldbank.org/DATASTATISTICS/Resources/GDP_PPP.pdf.

[53] Jacobs P, Calder P, Taylor M, Houston S, Saunders LD, Albert T (1999). Cost effectiveness of Streetworks' needle exchange program of Edmonton, Alberta.. http://www.ihe.ca/documents/1998-10paper.pdf.

[54] Health Protection Scotland (2010). Needle exchange surveillance initiative (NESI): Prevalence of HCV and injecting risk behaviours among injecting drug users attending needle exchanges in Scotland, 2008/2009..

[55] Vickerman P, Kumaranayake L, Balakireva O, Guinness L, Artyuck O (2006). The coste effectiveness of expanding harm reduction activities for injecting drug users in Odessa, Ukraine.. Sexually Transmitted Diseases.

[56] Vickerman P, Miners A, Williams J (2008). Assessing the cost-effectiveness of interventions linked to needle and syringe programmes for injecting drug users.. http://www.nice.org.uk/nicemedia/live/11829/40965/40965.pdf.

[57] NICE (2009). The guidelines manual 2009 - Chapter 7: Assessing cost effectiveness..

[58] Davis G, Beck J, Farrell G, Poynard T (1998). Prolonged treatment with interferon in patients with histologically mild chronic hepatitis C: A decision analysis.. Journal of Viral Hepatitis.

[59] Grieve R, Roberts J (2002). Economic evaluation for hepatitis C.. Acta Gastroenterolgica Belgica.

[60] NICE (2004). Guide to the methods of technology appraisal..

[61] Personal Social Services Research Unit (2009). Unit Costs of Health and Social Care 2009..

[62] Guinness L, Kumaranayake L, Rajaraman B, Sankaranaraya G, Vannela G (2005). Does scale matter? The costs of HIV-prevention interventions for commerical sex workers in India.. Bulletin of the World Health Organization.

[63] Guinness L, Kumaranayake L, Hansen K (2007). A cost function for HIV prevention services: Is there a ‘u’ - shape?. Cost Effectiveness and Resource Allocation.

[64] Johns B, Baltussen R (2004). Accounting for the cost of scaling-up health interventions.. Health Economics.

[65] Kumaranayake L (2008). The economics of scaling up: Cost estimation for HIV/AIDS interventions.. AIDS.

[66] DeAngelis D, Hickman M, Yang S (2004). Estimating long-term trends in the incidence and prevalence of opiate use/injecting drug use and the number of former users: Back-calculation methods and opiate overdose deaths.. American Journal of Epidemiology.

[67] Nanda S, Moore H, Lenhart S (2007). Optimal control of treatment in a mathematical model of chronic myelogenous leukemia.. Mathematical Biosciences.

[68] Panetta J, Fister K (2000). Optimal control applied to cell-cycle-specific cancer chemotherapy.. SIAM Journal on Applied Mathematics.

[69] Burden T, Ernstberger J, Fister K (2004). Optimal control applied to immunotherapy.. Discrete and Continuous Dynamical Systems Series B.

[70] Joshi H (2002). Optimal control of an HIV immunology model.. Optimal Control Applications and Methods.

[71] Kirschner D, Lenhart S, Serbin S (1997). Optimal control of the chemotherapy of HIV.. Journal of Mathematical Biology.

[72] Ledzewicz U, Schättler H (2007). Optimal controls for a model with pharmacokinetics maximizing bone marrow in cancer chemotherapy.. Mathematical Biosciences.

[73] Sutton AJ, Hope VD, Mathei C, Mravcik V, Sebakova H (2008). A comparison between the force of infection estimates for blood-borne viruses in injecting drug user populations across the European Union: A modelling study.. Journal of Viral Hepatitis.

[74] Nordt C, Stohler R (2006). Incidence of heroin use in Zurich, Switzerland: A treatment case register analysis.. The Lancet.

