# Supplementary figures and images for: Optimal Control of Hepatitis C Antiviral Treatment Programme Delivery for Prevention amongst a Population of Injecting Drug Users
Source: PLoS One. 2011 Aug 11;6(8):e22309. doi: 10.1371/journal.pone.0022309 (PMC3154900; doi:10.1371/journal.pone.0022309)

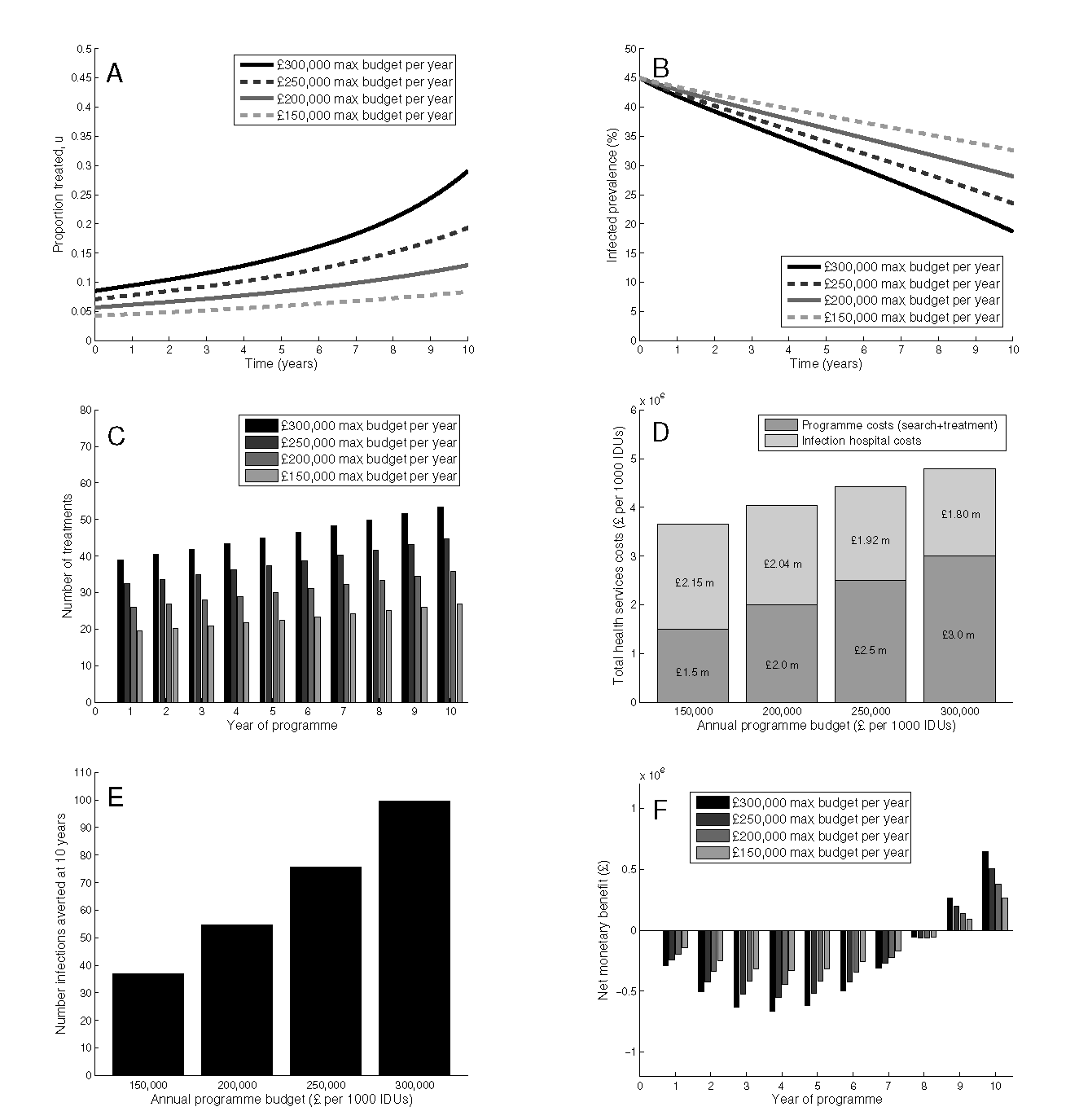

Supplement: Figure S1 — Scenario A: Minimising health service costs and HCV health utility losses. Simulations are with a 45% baseline prevalence, showing (A) programme coverage, (B) prevalence reductions, (C) number of treatments, (D) total health service costs (comprised of programme costs and infection costs), (E) infections averted, and (F) net monetary benefit. Parameters used are as shown in Tables 1–2, with , , , and with no final time prevalence target constraint. (TIFF) [file pone.0022309.s001.tif]

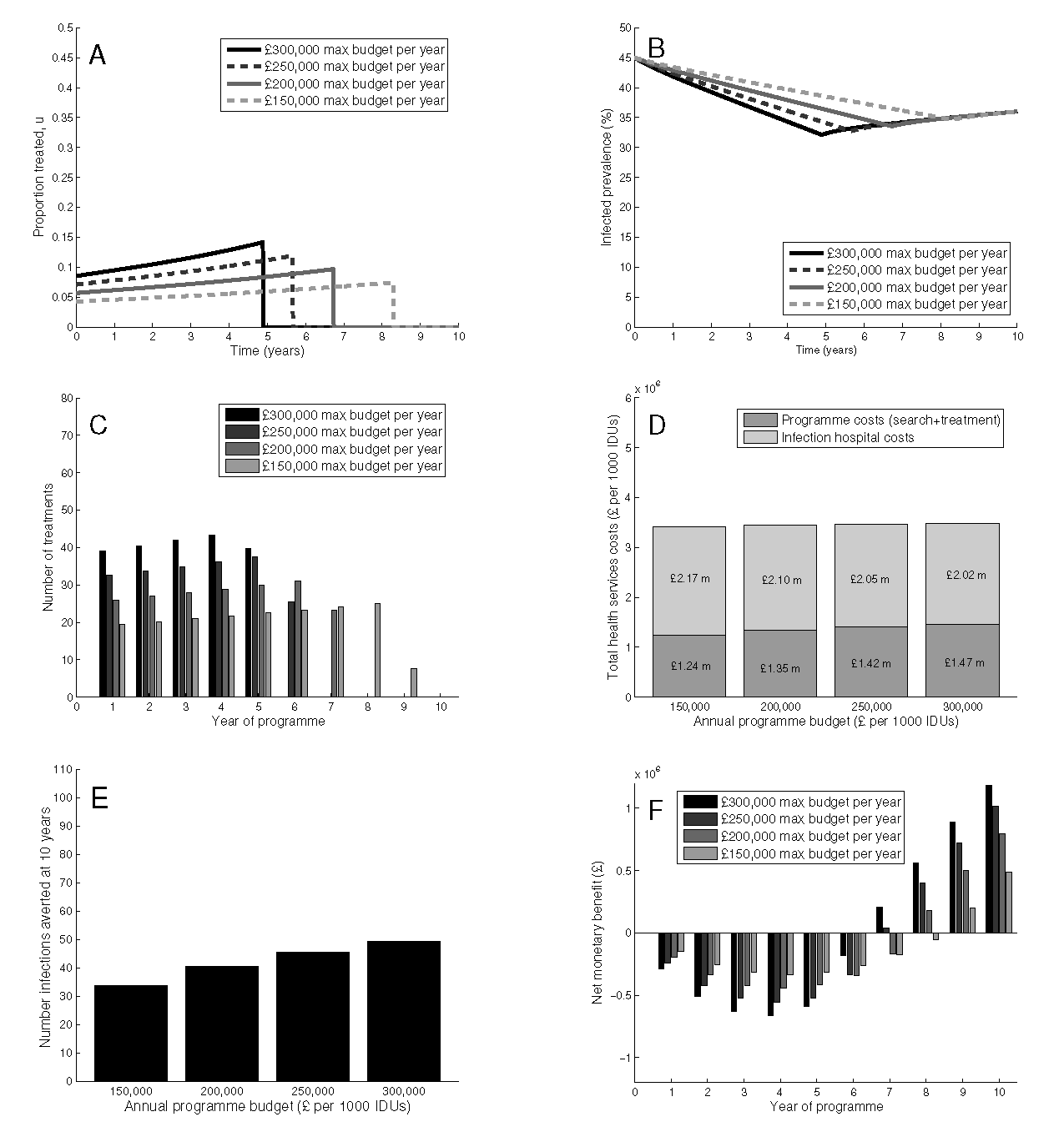

Supplement: Figure S2 — Scenario B: Minimising health service costs and HCV health utility losses with a final time prevalence target. Simulations are with a 45% baseline prevalence, showing (A) programme coverage, (B) prevalence reductions, (C) number of treatments, (D) total health service costs (comprised of programme costs and infection costs), (E) infections averted, and (F) net monetary benefit. Parameters used are as shown in Tables 1–2, with , , and a final time prevalence target constraint. (TIFF) [file pone.0022309.s002.tif]

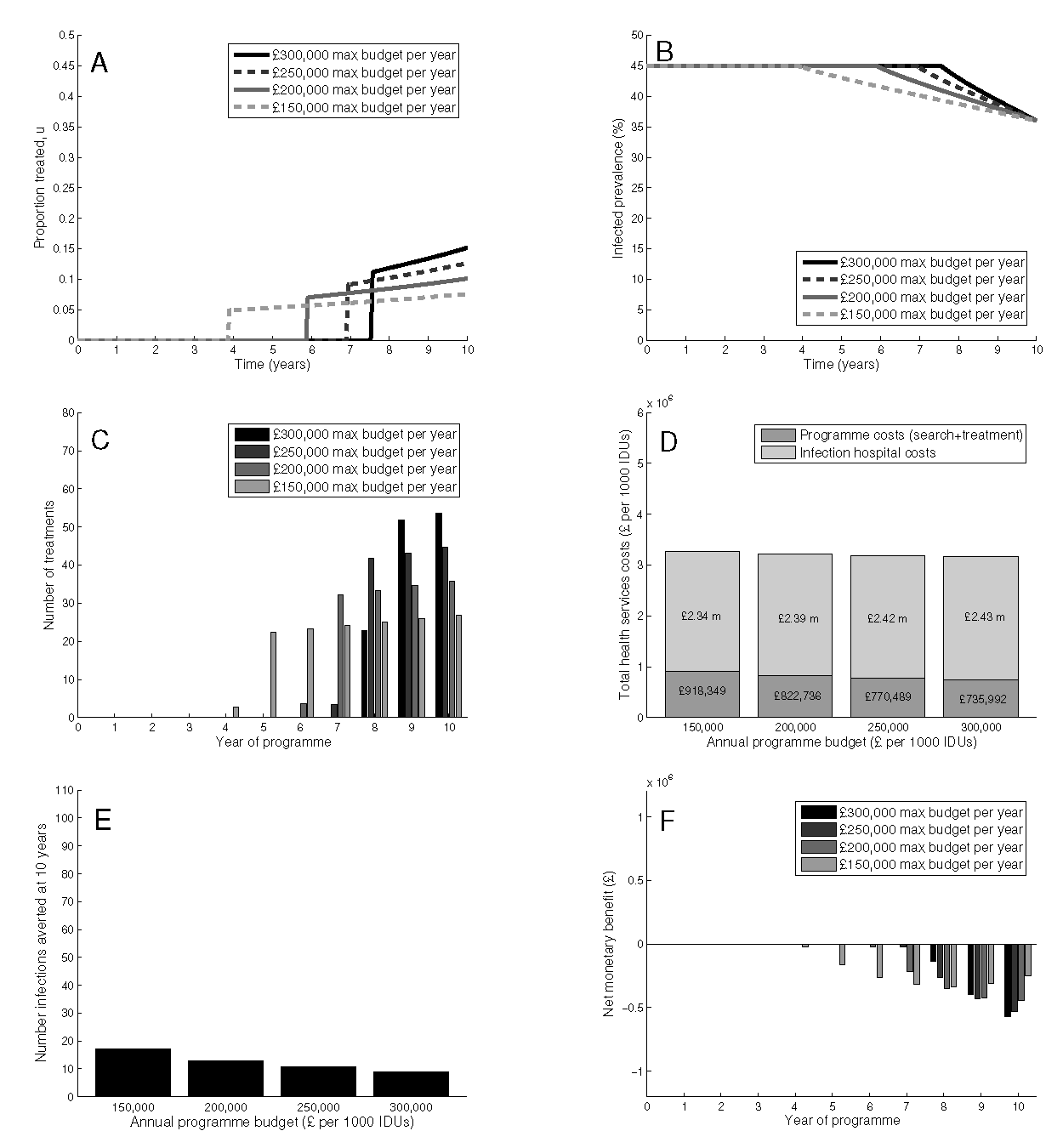

Supplement: Figure S3 — Scenario C: Minimising only health service costs with a final time prevalence target. Simulations are with a 45% baseline prevalence, showing (A) programme coverage, (B) prevalence reductions, (C) number of treatments, (D) total health service costs (comprised of programme costs and infection costs), (E) infections averted, and (F) net monetary benefit. Here, we neglect the health utility losses. Parameters used are as shown in Tables 1–2, with , , and a final time prevalence target constraint. (TIFF) [file pone.0022309.s003.tif]
